# From *N*-vinylpyrrolidone anions to modified paraffin-like oligomers via double alkylation with 1,8-dibromooctane: access to covalent networks and oligomeric amines for dye attachment

**DOI:** 10.3762/bjoc.12.133

**Published:** 2016-07-06

**Authors:** Daniela Obels, Melanie Lievenbrück, Helmut Ritter

**Affiliations:** 1 Heinrich-Heine University, Institute of Organic Chemistry and Macromolecular Chemistry, Universitaetsstraße 1, 40225 Duesseldorf, Germany

**Keywords:** double alkylation, modified *N*-vinylpyrrolidone, oligomeric anthraquinone dye, paraffin-like oligomer, radical thiol-ene click reaction

## Abstract

The double alkylation of *N*-vinylpyrrolidone (*N*-VP) with 1,8-dibromooctane yields paraffin-like oligomeric chains bearing polymerizable vinyl moieties. These oligomers were radically crosslinked in bulk with *N*-VP as co-monomer yielding swellable polymer disks. The vinylic side groups of the *N*-VP oligomers allow thiol–ene click reactions with 2-aminoethanethiol hydrochloride to obtain reactive amino-functionalized oligomers. Further modification of the free amino groups with 1,4-difluoro-9,10-anthraquinone (DFA) yields red-colored oligomeric anthraquinone dyes. The final reaction of DFA-substituted *N*-VP oligomers with Jeffamine^®^ M 600 leads to blue-colored and branched oligomers with poly(ethylene glycol) side chains.

## Introduction

Poly(*N*-vinylpyrrolidone) (PVP) is established in daily life due to its high water solubility and physiological compatibility [1–7]. It is used as additive, e.g., in cosmetics [8], pharmaceutical preparations [9–12] and food industry (additive E 1201) [13]. Monoalkylation reactions of *N*-VP in α-position via deprotonation by lithium diisopropylamide [14] were carried out to open up access to further potential applications of the corresponding polymers [14–16]. Thereby, the electron-rich double bond of *N*-VP is stable towards nucleophilic attack of the resulting carbanion intermediates. Some alkylated *N*-VP-based monomers can be used as agents in click chemistry [17] to obtain thermo- [18] or/and pH [19] responsive polymers. Besides classical alkylation reactions, Reinecke et al. performed ring-opening reactions to insert functional groups and aromatic side chains [20–21]. Furthermore, they investigated the formation of homogeneous [22] and amphiphilic [23] *N*-VP networks. However, in contrast to the restricted monoalkylation of *N*-vinylcaprolactam [24], the five-membered ring of *N*-VP can be double alkylated in α-position. In this connection, 1-bromo-2-(2-bromoethoxy)ethane was used as alkylation reagent to allow the preparation of a spiro-type monomer [25] due to intramolecular reaction. Until now, dibromo compounds were mainly used to synthesize symmetric cross linker [22,26–27]. To the best of our knowledge, the synthesis of modified paraffin-like oligomers via double alkylation of *N*-VP with aliphatic dibromides was not yet described in literature. In general, the term *paraffin* means *parum affinis* or poor reactivity and comprises acyclic alkanes. They are obtained as byproducts in petroleum industry [28–29]. Technical derivatives are chloro- and chlorosulfonated paraffins which are used, e.g., as surfactants [30–33].

In the current paper we wish to present the synthesis of paraffin-like oligomers via double alkylation of *N*-VP with 1,8-dibromooctane. Additionally, this work focuses on the use of the free double bonds for radical crosslinking as well as thiol-ene modification for subsequent dye attachment.

## Results and Discussion

**Scheme 1:**
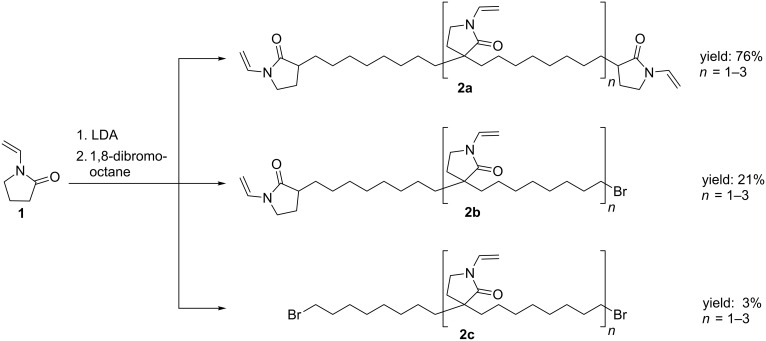
Alkylation of *N*-VP with 1,8-dibromooctane yielding paraffin-like oligomers **2a–c**.

*N*-Vinylpyrrolidone (**1**) can be mono or di-deprotonated in α-position to the carbonyl group by the use of the strong and sterically demanding base lithium diisopropylamide [14,34–35]. As mentioned above, the resulting carbanions do not attack the electron-rich *N*-vinyl double bond. Accordingly, by using 1,8-dibromooctane as alkylation reagent, extending the reaction time and working with a high concentrated solution a mixture of paraffin-like oligomers **2a–c** differing in their end groups was obtained. The oligomers were separated by use of column chromatography (Scheme 1).

The chemical structures of the derivatives **2a–c** were verified by ^1^H NMR spectroscopy and ESI mass spectrometry. Accordingly, a maximal number of three repeating units could be detected and the corresponding data are given in Supporting Information File 1.

**Figure 1:**
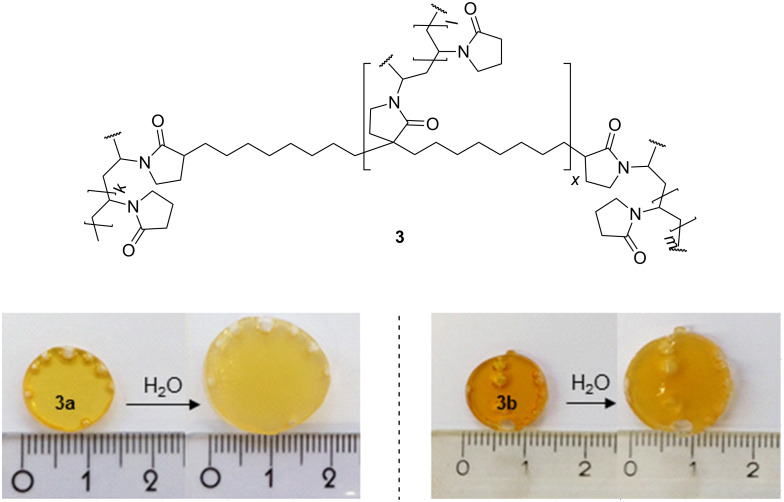
Chemical structure of water swellable network **3a** and **3b**. Photographs of water-swollen polymer disks consisting of 1 mol % (**3a**) and 2.5 mol % (**3b**) of **2a** as cross linker.

The vinylic groups of **2a** can easily be radically copolymerized in bulk with a molar excess of *N*-VP. By using either 1.0 mol % (Figure 1) or 2.5 mol % of **2a** in relation to *N*-VP, two different cross-linked polymers **3a**,**b** were obtained as disks showing a certain swelling behavior in distilled water (Figure 1).

The water uptake (W) [23,36] of the cross-linked polymers **3a** and **3b** in distilled water was followed gravimetrically. Equation 1 was used for the calculation,

[1]
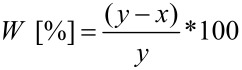


where *W* = water uptake, *x* = weight of dry polymer disk, and *y* = weight of swollen polymer disk.

As expected, the water uptake decreased with increasing the amount of used cross linker **2a** (Table 1) [37–39].

**Table 1:** Water uptake of cross-linked polymers **3a** and **3b**.

Polymer (disk)	Content of **2a** [mol %]	Water uptake [%]^a^

**3a**	1	189
**3b**	2.5	104

^a^Mean value of 3 measurements.

**Scheme 2:**
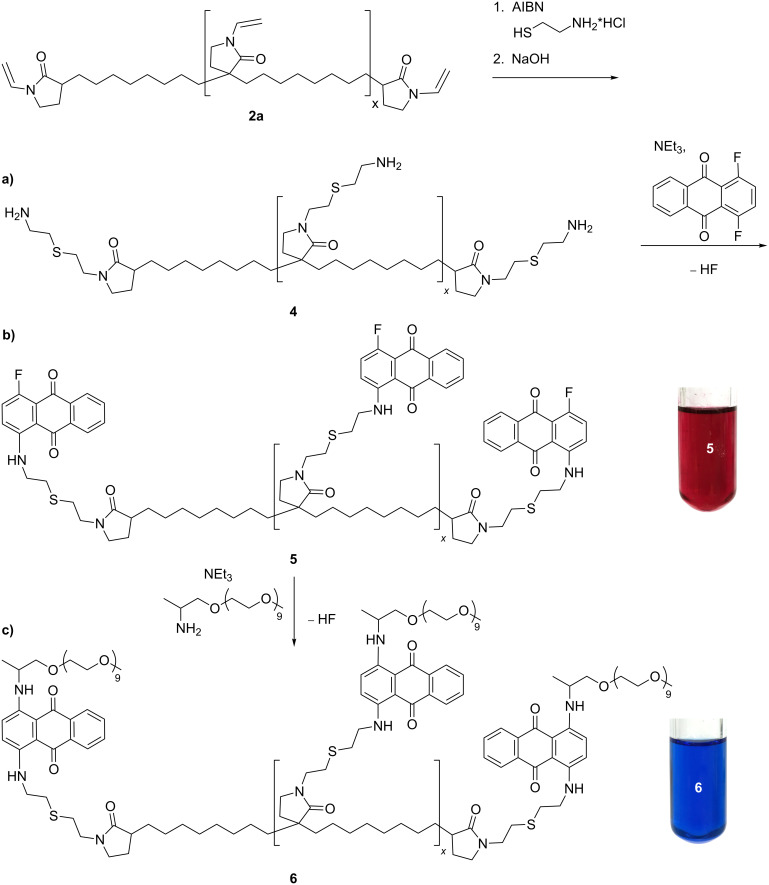
Synthesis of the branched oligomeric dye **6**: a) radical thiol–ene click reaction of **2a** with 2-aminoethanethiol hydrochloride yielding **4**. b) Subsequent reaction of **4** with DFA leading to red-colored oligomeric dye **5**. c) Branching of **5** with Jeffamine^®^ M 600 yielding blue-colored oligomer dye **6**.

Additionally, the free double bonds of **2a** were subjected to further modification through a thiol–ene [40–42] click reaction with 2-aminoethanethiol hydrochloride yielding oligomer **4** (Scheme 2). Subsequently, the reactivity of the primary amino groups in **4** was proven by the attachment of 1,4-difluoro-9,10-anthraquinone (DFA). Due to the strongly graduated reactivity of DFA, mono-functionalization is feasible, accompanied by a visible color change from yellow to red. This feature allows a reaction control with the naked eye and also with, e.g., UV–vis spectroscopy. The following reaction of DFA-substituted polymer **5** with Jeffamine^®^ M 600 leads to blue-colored branched oligomer **6** with poly(ethylene glycol) side chains. The conducted reaction sequence is shown in Scheme 2.

The quantitative conversion of the double bonds of oligomer **2a** was verified by ^1^H NMR spectroscopy. As described above, the corresponding product **4** bears reactive amino groups for further modifications. The successful reaction of **4** with DFA can be monitored by ^1^H NMR spectroscopy through the appearance of an aromatic NH signal at about 10 ppm and additionally on the change of the chemical shifts of the protons next to the fluoro substituents in attached DFA. The synthesis was carried out under mild conditions with a slight excess of DFA to achieve high conversions. Therefore, the ^1^H NMR and UV–vis spectra, respectively still showed signals of ca. 20 mol % of DFA [43] and the corresponding data are given in Supporting Information File 1.

**Figure 2:**
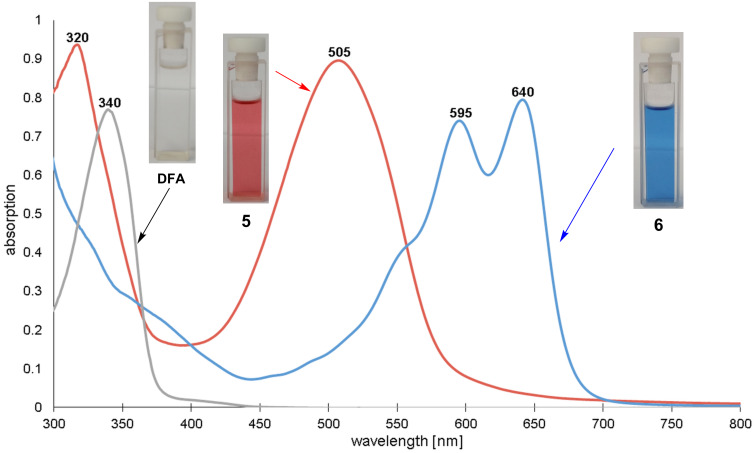
UV–vis spectra of oligomers **5** (*c* = 0.16 g/mL, red), **6** (*c* = 0.63 g/mL, blue) and DFA (*c* = 0.016 g/mL, grey).

The second fluoro substituent of the attached anthraquinone moiety in **5** was replaced at elevated temperature with *O*-(2-aminopropyl)-*O*'-(2-methoxyethyl)propylene glycol (Jeffamine^®^ M 600) yielding the branched blue-colored oligomeric dye **6**. The deep blue color is a result of the presence of two amino groups in 1,4-position of the anthraquinone dye [44]. Thus, this reaction can also be followed with the naked eye. Furthermore, the conversion of oligomer **6** was verified with UV–vis spectroscopy (Figure 2).

Accordingly, the different UV–vis spectra (Figure 2) of DFA, oligomer **5** and the disubstituted anthraquinone dye in **6**, respectively clearly verify the successful modification reactions. The complete conversion of **5** with Jeffamine^®^ M 600 is proven by the shift of the absorption maxima from 320 nm and 505 nm to higher wavelengths at 595 nm and 640 nm. According to literature, the introduction of amino groups in the 1- and 1,4-position of anthraquinones leads to a charge transfer of electrons from the amino group to the carbonyl functionality which results in additional π–π* absorption bands in the spectra of these compounds [45].

## Conclusion

The synthesis of paraffin-like oligomers **2a–c** via double alkylation of *N*-VP with 1,8-dibromooctane can easily be conducted through a one-pot synthesis. Swellable networks can be obtained by radical copolymerization of **2a** with *N*-VP. Furthermore, the conversion of the double bonds through thiol–ene click reaction with 2-aminoethanethiol hydrochloride leads to paraffinic oligomers **4** bearing primary amino groups. The reactivity of the latter functionalities was exemplarily demonstrated by the reaction with the dye 1,4-difluoro-9,10-anthraquinone resulting in the red-colored derivative **5**. Further reaction of **5** with Jeffamine^®^ M 600 yielded the blue-colored branched oligomer **6**. Both reactions can easily be monitored with UV–vis spectroscopy as well as with the naked eye. This work contributes to the increasing area of α-alkylation reactions of *N*-vinyl lactams and the subsequent application of the so obtained compounds to the synthesis of functionalized oligomers.

## Supporting Information

File 1 Experimental.
