# Supplementary material for: From N-vinylpyrrolidone anions to modified paraffin-like oligomers via double alkylation with 1,8-dibromooctane: access to covalent networks and oligomeric amines for dye attachment
Source: Beilstein J Org Chem. 2016 Jul 6;12:1395–400. doi: 10.3762/bjoc.12.133 (PMC4979690; doi:10.3762/bjoc.12.133)
Supplement: File 1 — Experimental. [file Beilstein_J_Org_Chem-12-1395-s001.pdf]

Supporting Information

for

From *N*-vinylpyrrolidone anions to modified paraffin-like oligomers via double alkylation with 1,8-dibromooctane: access to covalent networks and oligomeric amines for dye attachment

Daniela Obels, Melanie Lievenbrück and Helmut Ritter*

Address: Heinrich-Heine University, Institute of Organic Chemistry and Macromolecular Chemistry, Universitaetsstraße 1, 40225 Duesseldorf, Germany

Email: Helmut Ritter - h.ritter@uni-duesseldorf.de

*Corresponding author

Experimental

Content:

Experimental section

- Materials
- Measurements
- Synthesis

Analyses

- ^{1}H NMR spectra
- IR spectra
- Electrospray ionization spectra

References

Experimental section

- Materials

Lithium diisopropylamide (2 M solution in hexane/*n*-heptane/ethylbenzene), hydrochloric acid (fuming, 37%) and *O*-(2-aminopropyl)-*O*`-(2-methoxyethyl)propylene glycol (Jeffamine M 600) were purchased from Sigma–Aldrich (Germany). *N*-Vinylpyrrolidone (99%), 1,8-dibromooctane (98%), silica gel (for column chromatography ultra pure, 40–60 µm, 60Å) and 2,2'-azobis(2-methylpropionitrile) (98%) were obtained from Acros Organics (Belgium). *N*-Vinylpyrrolidone was distilled before use. 2-Mercaptoethylamine hydrochloride (98%) was purchased from Alfar Aesar (Germany). Sodium hydroxide (min 98.8%, p.a.) was obtained from ChemSolute (Germany). Sea sand p.a. and triethylamine (pure) were obtained from AppliChem (Germany). Toluene, methanol und dimethyl sulfoxide (all analytic reagent grade) were purchased from Fischer Chemicals (Germany). Deuterated dimethyl sulfoxide (DMSO-d_6) and deuterated chloroform ($CDCl_3$) for spectroscopy were obtained from deutero GmbH (Germany). Tetrahydrofuran was distilled, stored over molecular sieve (4 Å) and used until 14 days after distillation. Unless otherwise noted, all operations were performed under an inert atmosphere using dried glassware. The synthetic apparatus used was a three-necked or a one-necked flask, equipped with magnetic stirrer and argon inlet.

- Measurements

The structures of the oligomers are verified by ^{1}H NMR spectroscopy using a Bruker Avance III spectrometer at 300 MHz. The samples were measured at room temperature. When using DMSO-d_6 as solvent for ^{1}H NMR measurement, the chemical shifts were referenced to the residual solvent signal at δ = 2.5 ppm. Trimethylsilane was used as internal standard. Infrared spectra were recorded on a Nicolet 6700 FTIR spectrometer equipped with a

diamond single bounce ATR accessory at room temperature. Electrospray ionization (ESI) as well as electron ionization (EI) mass spectrometry was performed on an Ion-Trap-API-mass spectrometer from Finnigan LCQ Deca (Thermo Quest) in a range up to *m/z* 4000. The samples were dissolved in methanol or chloroform. Matrix-assisted laser desorption ionization time-of-flight (MALDI-TOF) mass spectrometry was performed on a Bruker Ultraflex mass spectrometer using a 337 nm nitrogen laser. An adequate sample matrix was used and the samples were dissolved in methanol. UV–vis spectroscopic measurements were performed using a dual-trace spectrometer Specord® 210 Plus from Analytik Jena AG. The solutions were placed in a quartz cuvette after being passed through a 0.45 µm filter to remove particle impurities. The measurements were performed at room temperature between 300 and 800 nm.

- **Synthesis**

Oligomers 2a–c

Analogously as described in reference [25], the equipment was heated and purged with argon to achieve water-free conditions. A solution of 4.3 mL (40 mmol) *N*-vinylpyrrolidone (*N*-VP, **1**) dissolved in 5 mL abs. THF was added dropwise at −78 °C within 30 min to a solution of 40 mL (80 mmol) 2 M LDA. The mixture was stirred 1 h at −78 °C. Afterwards, 7.4 mL (40 mmol) of 1,8-dibromooctane in 5 mL abs. THF were added dropwise within 30 min. The reaction mixture was stirred at −78 °C for 1 h and subsequently 96 h at rt. After the addition of 50 mL dist. water and 45 mL dichloromethane the aqueous phase was extracted with dichloromethane (3 x 50mL). The organic phases were combined and the solvent was removed in vacuum. The viscous crude products were dried until constant weight, dissolved in methanol, and purified by column chromatography to give 8.8 g (yield

57%) of an oligomer mixture. To separate oligomers **2a**–**c** the mixture was subjected to further column chromatography. First, ethyl acetate was used as eluent to separate oligomers **2b** and **2c** yielding 2.8 g (yield 32%) of oligomer **2b** and 0.6 g (yield 7%) of oligomer **2c**. Then elution was continued using methanol to obtain 5.4 g of oligomer **2a** (yield 61%). All oligomers were highly viscous and brown substances.

^{1}H NMR Oligomer **2a** (300 MHz, $CDCl_3$): δ = 7.22-6.98 (m, 1H, CH_2-C**H**-N), 4.61-4.05 (m, 2H, C$\mathbf{H_2}$-CH-N), 3.63-3.27 (m, 2H, CH_2-C$\mathbf{H_2}$-N),2.74-2.33 (m, 1H, C$\mathbf{H_2}$-CH_2-N), 2.06-1.78 (m, 1H, C$\mathbf{H_2}$-CH_2-N), 1.57-1.06 (m, alkyl) ppm. MALDI-TOF (Oligomer **2a**): *m/z* 775 [4xVP], 997 [5xVP], 1218 [6xVP], 1439 [7xVP], 1661 [8xVP], 1882 [9xVP], 2108 [10xVP] FT-IR (Oligomer **2a**) (diamond): $\tilde{\nu}$ [cm^{-1}] = 2928/2855 (ν, CH_2, CH, w), 1694 (ν, amid I, C=O, s), 1629 (ν, amid II, NH, s), 1423 (δ, CH ring, m), 1385 (δ, CH alkyl, s), 1261 (ν, C-N, s). ESI (Oligomer **2a**): *m/z* 333 [2xVP], 555 [3xVP], 776 [4xVP], 997 [5xVP]

ESI (Oligomer **2b**): *m/z* 303 [1xVP, 1xBr), 495 [1xVP, 2xBr], 716 [2xVP, 2xBr], 937 [3xVP, 2xBr].

ESI (Oligomer **2c**): *m/z* 304 [1xVP+Br], 526 [2xVP+Br], 747 [3xVP+Br], 968 [4xVP+Br]

Cross-linked blank 3

blank	ratio X of oligomer 2a	AIBN (1 mol%)
**1 mol%**	288 mg	32.3 mg
**2.5 mol%**	570 mg	33.8 mg

X mg of oligomer **2a** were dissolved in 2 mL (0.019 mol) of **1** and purged with argon for 10 min. Afterwards, 1 mol % of 2,2’-azobis(2-methylpropionitrile) (AIBN) as radical starter

was added and the mixture was heated to 60 °C to obtain clear and cross-linked yellow discs. The water uptake capability of the obtained discs in distilled water was investigated.

**disc**		**weight before [mg]**	**weight after [mg]**	**water uptake [mg];[%]**	**Ø [%]**
**1 mol %**	1.	266.91	780.16	1.93; 193	
	2.	253.67	749.44	1.95; 195	189
	3.	271.31	758.63	1.79; 179	
**2.5 mol %**	1.	254.17	511.84	1,01; 101	
	2.	237.75	498.53	1,09; 109	104
	3.	249.06	506.08	1.03; 103	

The water uptake was followed until constant weight. Therefore, a sample with 1% and a sample with 2.5% of oligomer **2a** were weighted and given into a small glass tank filled with 4 mL of distilled water. After 24 h the swollen samples were weighted again and the water was tested for residual monomers. The mass of the swollen polymer network compared to the mass of the dry network was determined using the following equation:

$$W = \frac{(y-x)}{y}$$

W = water uptake

x = weight of the unswollen disk

y = weight of the swollen disk

Oligo-amine 4

Oligomer **2a** (3.12 g, 5.634 mmol) was dissolved in 4 mL toluene and purged with argon for 10 min. Afterwards, 0.96 g (1.5 mol %) cysteamine hydrochloride and 0.0925 g (10 mol %) of 2,2'-azobis(2-methylpropionitrile) as radical starter were added. The mixture was heated to 65 °C bath temperature for 72 h. After that the reaction mixture was diluted with 4 mL methanol and dried in vacuum. The crude product was dissolved in water acidified with

hydrochloric acid to transfer the primary amine groups into the water soluble corresponding salt. Sodium hydroxide was added to the filtered solution to obtain the oligomer with primary amino groups. Afterwards, the solvent was removed and the product was dried in vacuum again.

^{1}H NMR Oligomer **4** (300 MHz, dmso-d_6): δ = 7.22-6.98 (m, 1H, CH_2-C**H**-N), 4.61-4.05 (m, 2H, C**H₂**-CH-N), 3.63-3.27 (m, 2H, CH_2-C**H₂**-N),2.74-2.33 (m, 1H, C**H**$_2$-CH_2-N), 2.06-1.78 (m, 1H, C**H**$_2$-CH_2-N), 1.57-1.06 (m, alkyl) ppm. FT-IR (diamond): $\tilde{\nu}$ [cm^{-1}] = 3360 (m, NH_2, m), 2929/2851 (*v*, CH_2, CH, w), 1668 (*v*, amid I, C=O, s), 1631 (*v*, amid II, NH, s), 1424 (δ, CH ring, m), 1390 (δ, CH alkyl, s), 1268 (*v*, C-N, s).

1,4-difluoro-9,10-anthraquinone (DFA)

1,4-Difuoro-9,10-anthraquinone was prepared according to a method described in literature.[1] The crude product was obtained as a yellow solid (yield: 60%) and purified via column chromatography (dichlormethane/hexane 2:1).

^{1}H NMR DFA (300 MHz, DMSO-d_6): δ = δ 8.13 (dd,^{3}J=5.8 Hz, ^{4}J=3.3 Hz, 2H, ArH), 7.92 (dd, ^{3}J=5.8 Hz, ^{4}J=3.3 Hz, 2H, ArH), 7.84-7.77 (m, 2H, ArH) ppm. FT-IR (diamond): $\tilde{\nu}$ [cm^{-1}] = 3084 (υ, C=C, s) 1678 (υ, C=O, s), 1587 (υ, C=C, m), 1249 (υ, C-F, vs), 719 (δ, C-F, s). EI: *m/z 244.*

Oligo-anthraquinone dye 5

Oligomer **4** (0.1 g, 0.0595 mmol) and 0.131 g (0.2678 mmol) of 1,4-difluoro-9,10-anthraquinone were separately dissolved in 1 mL of dimethyl sulfoxide and combined. To keep a basic surrounding, 0.5 mL triethylamine were added. After a few minutes a color change of the reaction mixture can be recognized. To complete the reaction

the mixture was stirred for 24 h. Afterwards the product was dried in vacuum until constant weight.

^{1}H NMR oligomer 5 (300 MHz, DMSO-d$_6$): δ = 9.97 (s, 1H,C-N**H**-CH$_2$), 8.11 (m, J = 10.8 Hz, 2H, C**H**-CH-C, ArH), 7.67 – 7.52 (m, 2H, CH-C**H**-C, ArH), 7.22 (d, J = 12.0 Hz, 1H, CF-C**H**, ArH), 7.02 (s, 1H, CNH-C**H**, ArH), 3.43 (d, J = 24.3 Hz, 4H, C**H$_2$**-N-C**H$_2$**), 3.32 – 3.07 (m, 2H, NH-C**H$_2$**-CH$_2$), 2.89 – 2.75 (m, 2H, NH-C**H$_2$**-S-CH$_2$), 2.74 – 2.58 (m, 2H, NH-CH$_2$-S-C**H$_2$**), 2.27 (s, 1H, C**H**$_2$-CH$_2$-N), 2.06 (s, 1H, C**H**$_2$-CH$_2$-N), 1.85 – 0.83 (m, alkyl). FT-IR (diamond): $\tilde{\nu}$ [cm^{-1}] = 3401 (m, NH, m), 2925/2853 (ν, CH$_2$, CH, w), 1667 (ν, amid I, C=O, s), 1633 (ν, amid II, NH, s), 1593/1509 (υ, C=C, m), 1434 (δ, CH ring, m), 1261 (ν, C-N, s), 1242 (υ, C-F, vs), 723 (δ, C-F, s).

Oligo-Anthraquinone dye 6

Oligomer **5** (0.6 g, 0,3 mmol) was dissolved in 0.5 mL of dimethyl sulfoxide and combined with 1.48 mL (0.24mmol) of Jeffamin M 600. To keep a basic surrounding 0.5 mL triethylamine were added. Under constant stirring, the reaction mixture was heated to 70 °C (oil bath) and after 24 h a color change of the reaction mixture can be recognized. To complete the reaction the mixture was stirred for additional 96 h. Afterwards the product was dried in vacuum until constant weight.

FT-IR (diamond): $\tilde{\nu}$ [cm^{-1}] = 3424 (m, NH, m), 2971/2927/2869 (ν, CH$_2$, CH, w), 1434 (δ, CH ring, m), 1261 (ν, C-N, s), 1373 (υ, CH$_3$, m), 1094 (δ, C-O-C, s).

- **Analysis**

^{1}H NMR spectra

The spectrum of oligomer **2a** shows the unmodified double bonds at 4.4 ppm (CH_2, blue box) and the down field shift of the proton next to the nitrogen atom at 6.9 ppm (CH, orange box).

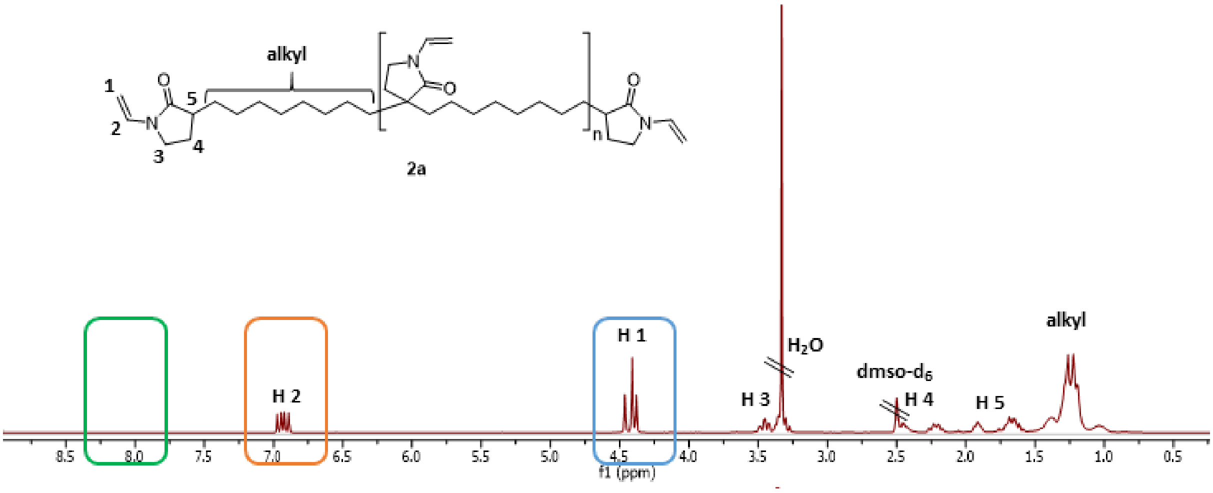

*Figure S1. ^{1}H NMR spectrum of oligomer **2a** in DMSO-d_6.*

Figure S2 clearly shows the synthesis of oligo-amine **4**. No signal of the double bounds at 4.4 ppm (blue box) or 6.9 ppm (orange box) can be noticed. An additional amine signal is present at 8.1 ppm (green box).

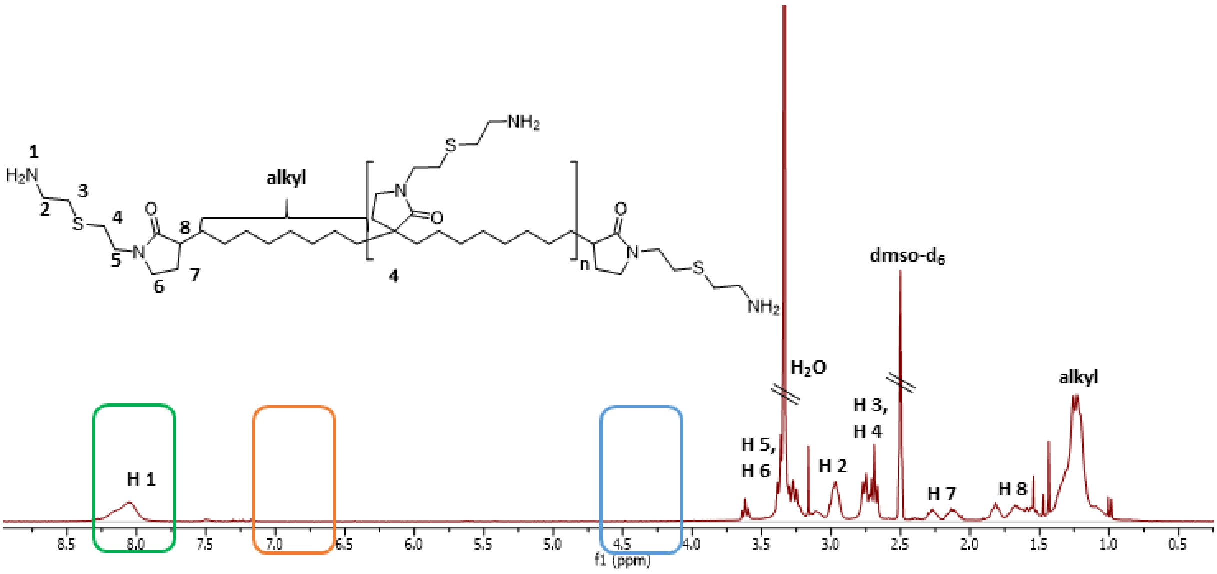

Figure S2. ^{1}*H NMR spectrum of oligomer* ***4*** *in DMSO-d*$_6$.

Figure S3 shows the spectrum of DFA. Based on the symmetric structure, the protons of this compound generate three peaks in the corresponding spectrum.

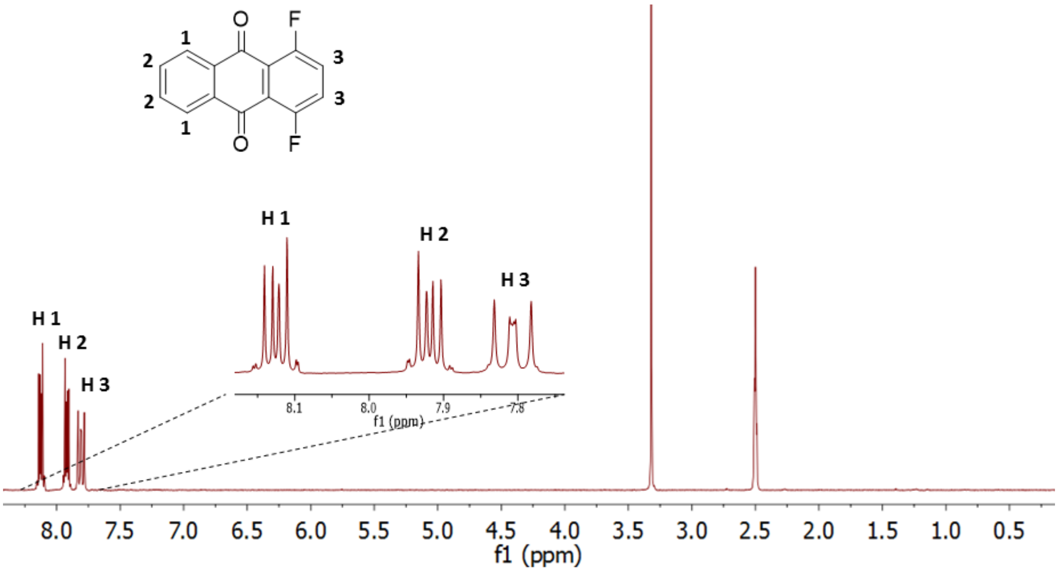

Figure S3. ^{1}*H NMR spectrum of* ***DFA*** *in DMSO-d*$_6$.

Figure S4 shows the spectrum of the synthesized oligomeric red dye **5**. As a result of the chemical bonding of DFA the spectrum shows the down field shift of the amino group to 9.9 ppm. Furthermore a split up of the peaks next to the flouro substituent can be observed.

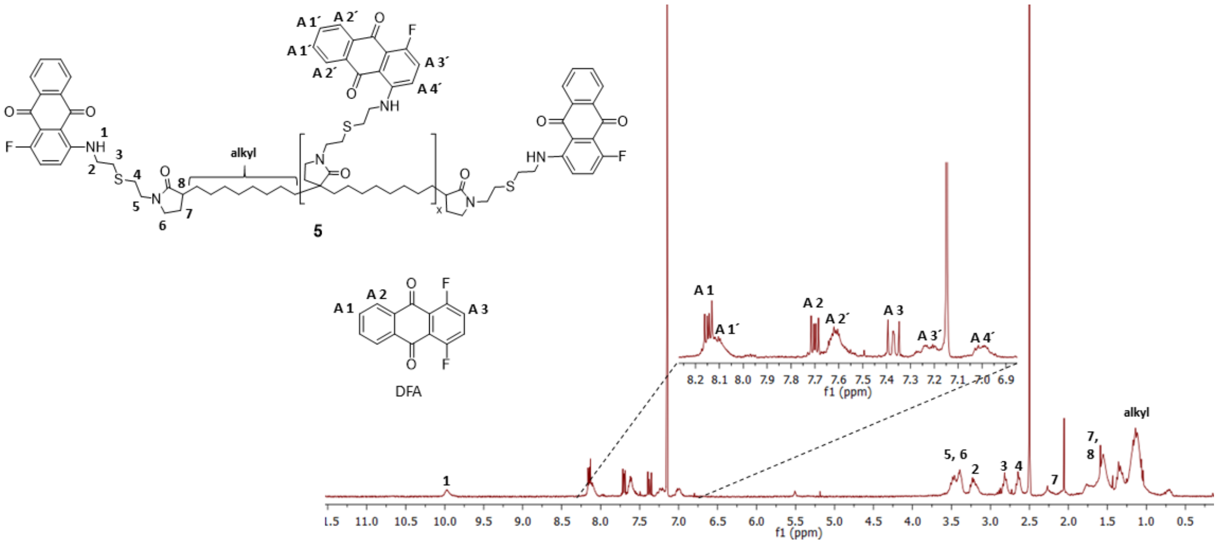

*Figure S4. ^{1}H NMR spectrum of oligomer **5** in DMSO-d$_6$.*

IR spectra

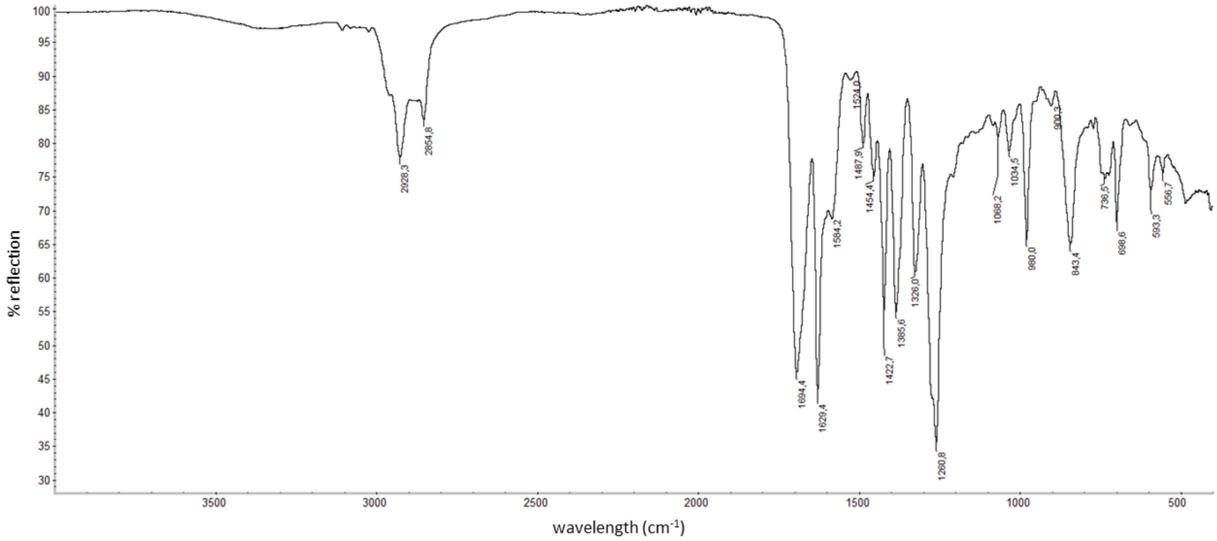

*Figure S5. IR spectrum of oligomer **2a**.*

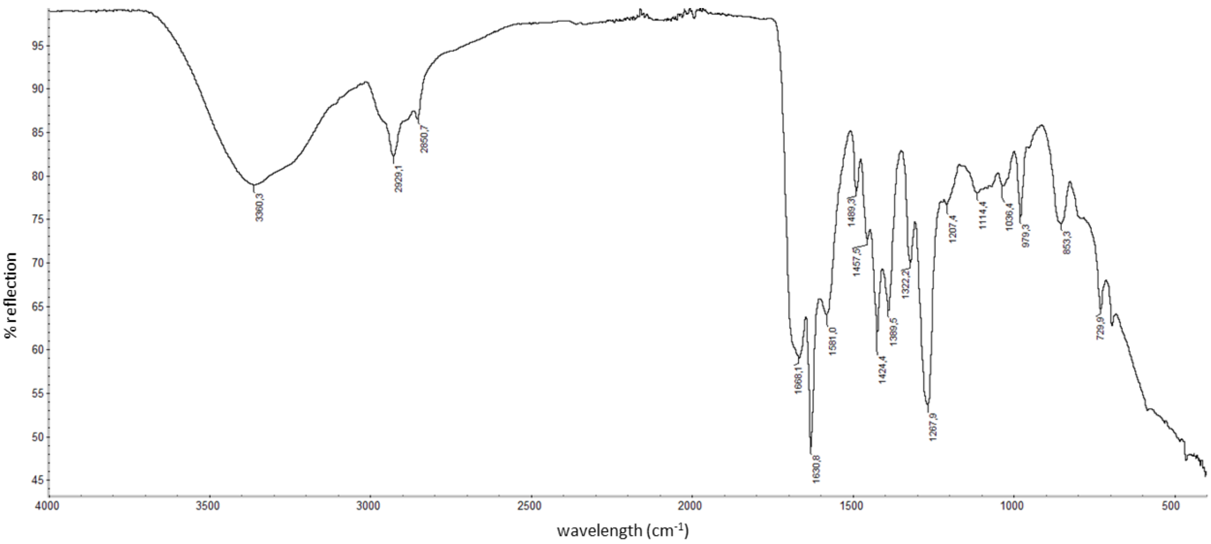

*Figure S6. IR spectrum of oligomer **4**.*

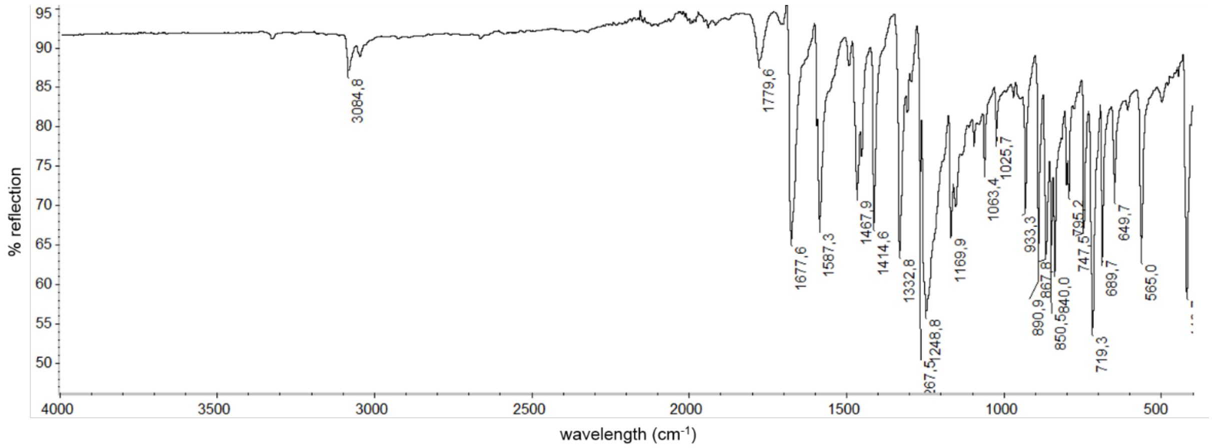

*Figure S7. IR spectrum of 1,4-difuoro-9,10-anthraquinone (**DFA**).*

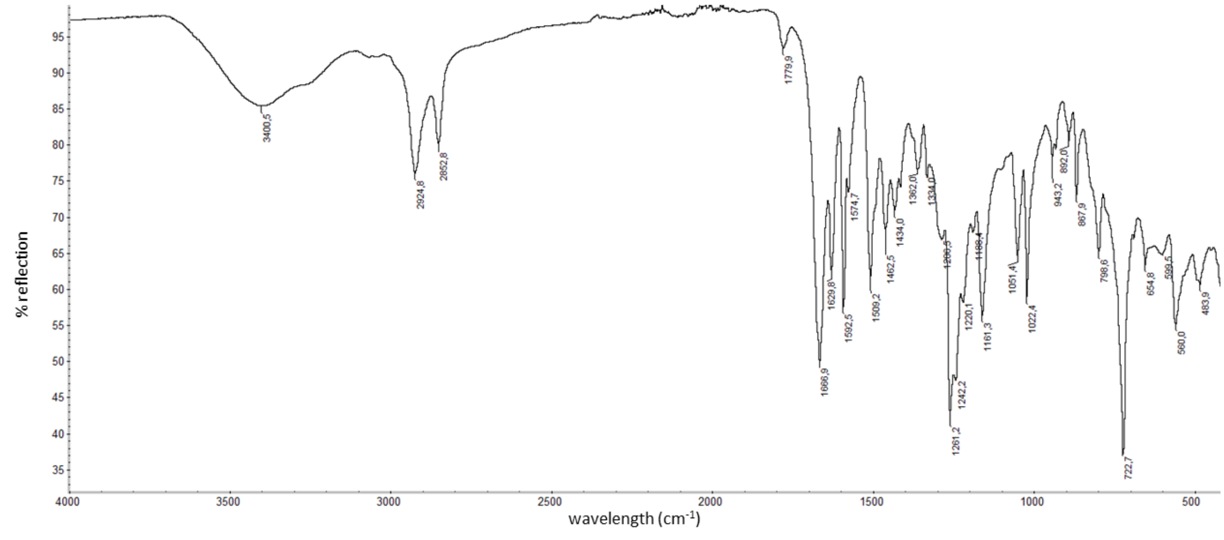

*Figure S8. IR spectrum of oligomer **5**.*

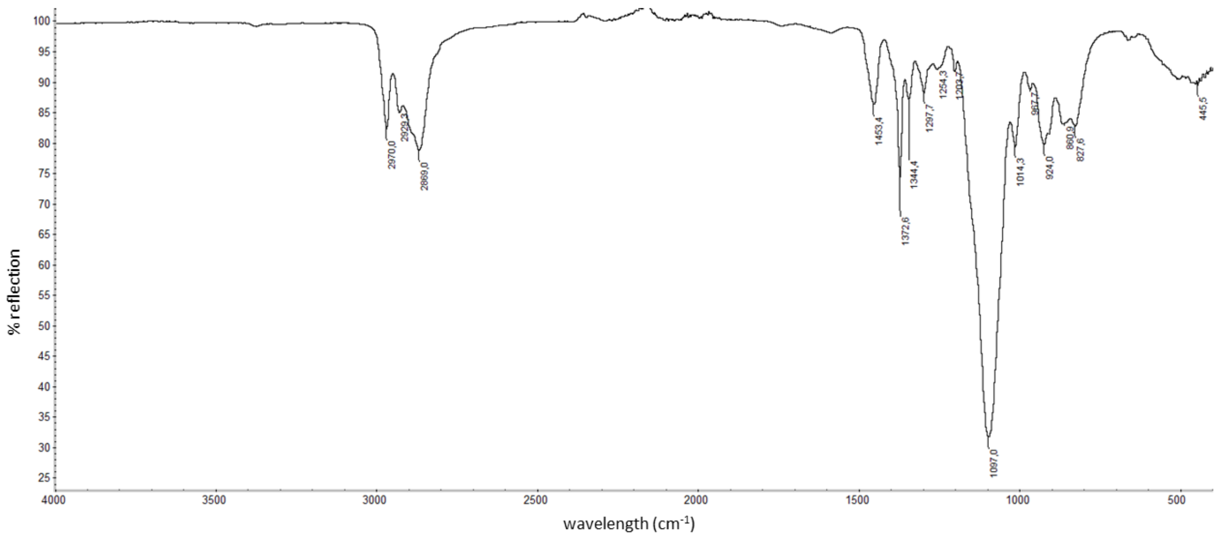

Figure S9. IR spectrum of Jeffamine M 600.

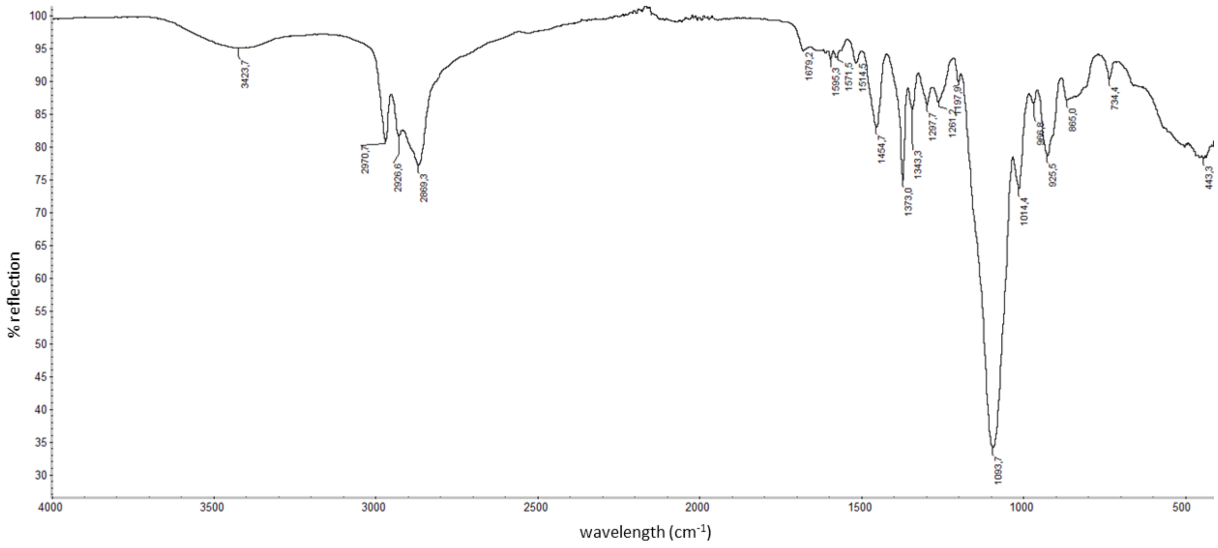

*Figure S10. IR spectrum of Oligomer **6**.*

ESI spectra

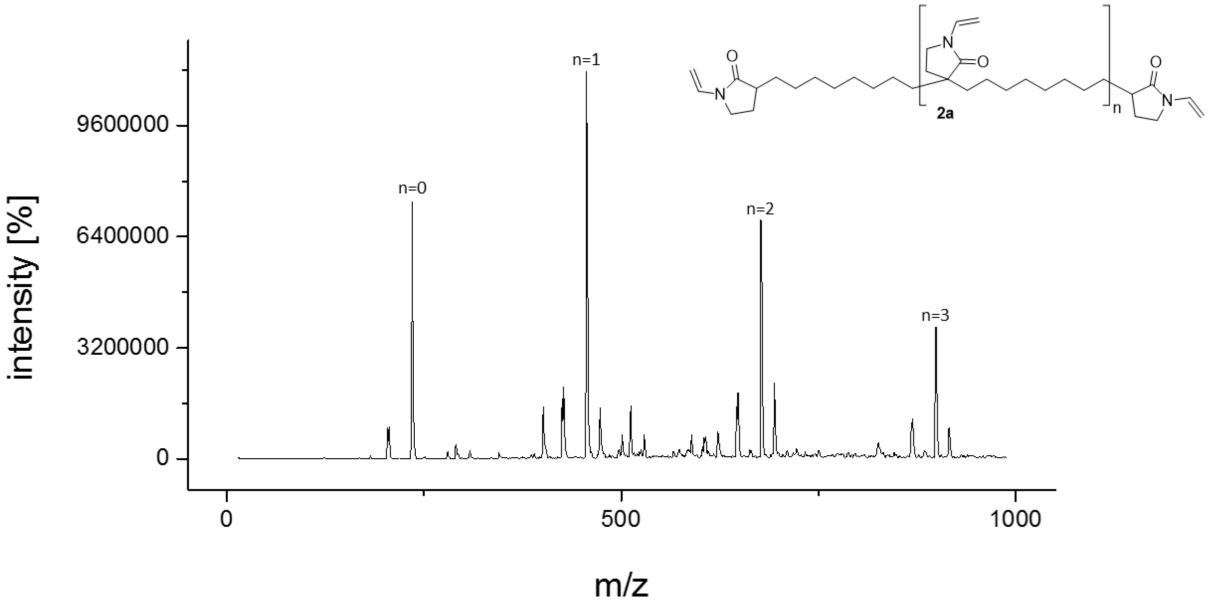

*Figure S11. ESI spectrum of oligomer **2a***

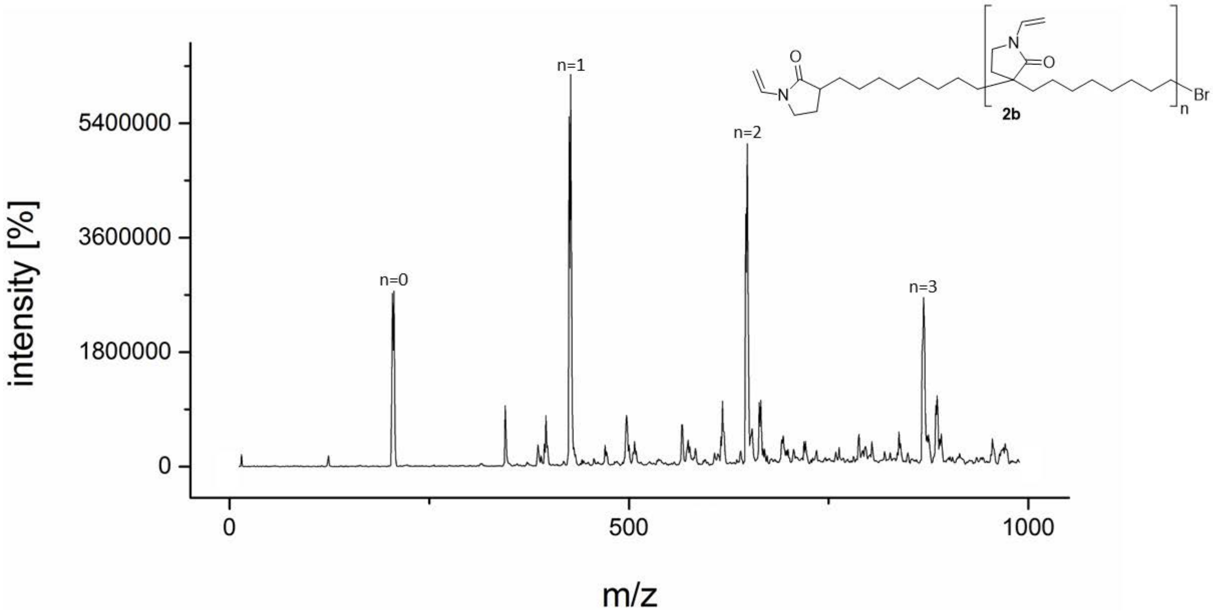

*Figure S12. ESI spectrum of oligomer **2b**.*

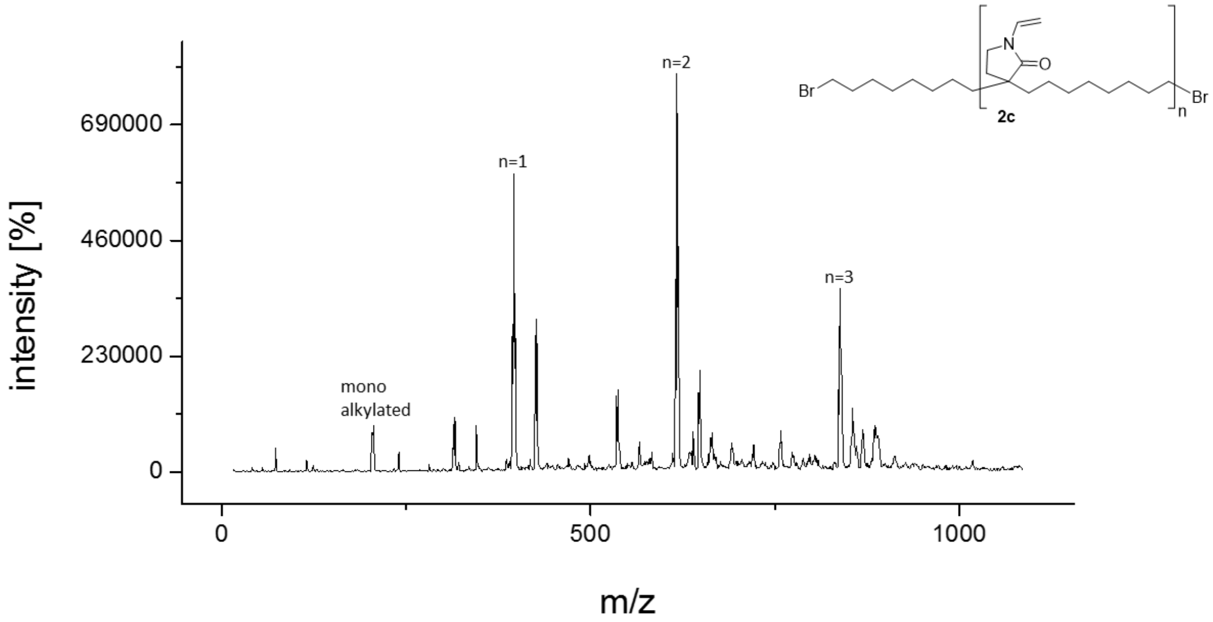

*Figure S13. ESI spectrum of oligomer **2c**.*

UV–vis Spectra:

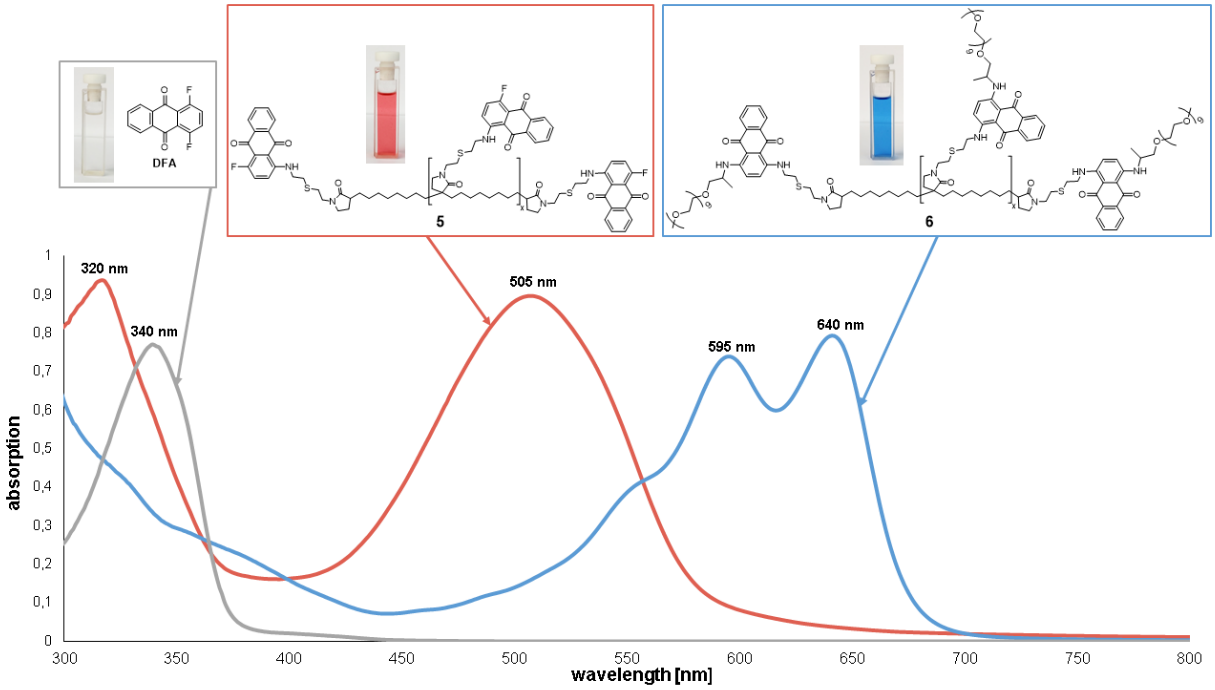

*Figure S14. UV–vis spectra of oligomers **5**, **6** and **DFA**.*

- ***References***

[1] A. P. Krapcho, Z. Getahun, *Synthetic Communications* **1985**, *15*, 907-910.
